# Predictability alters multisensory responses by modulating unisensory inputs

**DOI:** 10.3389/fnins.2023.1150168

**Published:** 2023-03-29

**Authors:** Scott A. Smyre, Naomi L. Bean, Barry E. Stein, Benjamin A. Rowland

**Affiliations:** Department of Neurobiology & Anatomy, Wake Forest School of Medicine, Winston-Salem, NC, United States

**Keywords:** multisensory, cross-modal, superior colliculus, prediction, attenuation, potentiation

## Abstract

The multisensory (deep) layers of the superior colliculus (SC) play an important role in detecting, localizing, and guiding orientation responses to salient events in the environment. Essential to this role is the ability of SC neurons to enhance their responses to events detected by more than one sensory modality and to become desensitized (‘attenuated’ or ‘habituated’) or sensitized (‘potentiated’) to events that are predictable *via* modulatory dynamics. To identify the nature of these modulatory dynamics, we examined how the repetition of different sensory stimuli affected the unisensory and multisensory responses of neurons in the cat SC. Neurons were presented with 2HZ stimulus trains of three identical visual, auditory, or combined visual–auditory stimuli, followed by a fourth stimulus that was either the same or different (‘switch’). Modulatory dynamics proved to be sensory-specific: they did not transfer when the stimulus switched to another modality. However, they did transfer when switching from the visual–auditory stimulus train to either of its modality-specific component stimuli and vice versa. These observations suggest that predictions, in the form of modulatory dynamics induced by stimulus repetition, are independently sourced from and applied to the modality-specific inputs to the multisensory neuron. This falsifies several plausible mechanisms for these modulatory dynamics: they neither produce general changes in the neuron’s transform, nor are they dependent on the neuron’s output.

## 1. Introduction

The superior colliculus (SC) is a multisensory midbrain structure particularly well suited to its role in rapidly processing sensory information to facilitate detection, localization, and orientation to salient environmental events (Stein and Meredith, 1993). Many of its deep layer neurons receive inputs from multiple senses, with their different receptive fields in spatial register, thereby giving the structure three overlapping topographic sensory representations (Stein et al., 1975; Knudsen, 1982; Palmer and King, 1982; Jay and Sparks, 1984; Middlebrooks and Knudsen, 1984). As a result, stimuli derived from the same environmental event, regardless of modality, activate the same SC region. And, because these neurons can synthesize their different sensory inputs to amplify their responses, they can increase the physiological salience of that event and its likelihood of being detected and localized (Hartline et al., 1978; Meredith and Stein, 1983, 1986a,b; King and Palmer, 1985; Stein et al., 1989; Wallace et al., 1998; Burnett et al., 2004; Perrault et al., 2005; Stanford et al., 2005; Alvarado et al., 2007; Rowland et al., 2007a,b,c; Rowland and Stein, 2008; Zahar et al., 2009). Because these neurons project to motor areas of the brainstem and spinal cord, they can also initiate appropriate orientation responses to that event (Sprague and Meikle, 1965; Stein and Clamann, 1981; Huerta and Harting, 1982; McHaffie and Stein, 1982; Meredith and Stein, 1985; Sparks, 1986; Stein et al., 1989; Meredith et al., 1992; Wallace et al., 1993; Frens and Van Opstal, 1998; Burnett et al., 2004, 2007).

Consistent with their behavioral role, SC neurons are sensitive to stimulus predictability. Their responses can be attenuated by rapid repetitions of the same innocuous stimulus, a phenomenon often referred to as “habituation” (Harutiunian-Kozak et al., 1971; Stein et al., 1973a,b, 1976; Oyster and Takahashi, 1975; Chalupa and Rhoades, 1977; Woods and Frost, 1977; Fecteau and Munoz, 2005; Reches and Gutfreund, 2008; Boehnke et al., 2011; Netser et al., 2011; Dutta and Gutfreund, 2014). This minimizes responses to inconsequential “background” stimuli, decreasing their likelihood of SC-mediated behaviors. This attenuation can be reversed immediately (“dishabituation”) by making changes that render the stimulus unpredictable; e.g., by altering stimulus features (e.g., see Sokolov, 1963; Reches and Gutfreund, 2008) or context (e.g., see Hoffman and Stitt, 1969; Boehnke et al., 2011). In addition, repeated stimulation at other rates and intensities can induce the opposite effect and potentiate responses (Hughes et al., 1956; Zhang et al., 2000; Perrault et al., 2011; Kordecka et al., 2020). This feature (often described as “neural facilitation”) allows signals to enhance their salience and recruit SC-mediated behaviors. In both cases, the response changes induced by stimulus predictability have obvious survival value, and it is important to understand how the modulatory dynamics producing these changes are sourced and applied.

Response attenuation/habituation has been extensively studied in SC neurons, typically by testing their responses to rapid trains of repeated visual (Harutiunian-Kozak et al., 1971; Woods and Frost, 1977; Boehnke et al., 2011), auditory (Reches and Gutfreund, 2008; Netser et al., 2011), or somatosensory (Stein et al., 1976; Castro-Alamancos and Favero, 2016) stimuli. However, because these studies only tested one sensory modality at a time, how the modulatory dynamics inducing these response changes are sourced and applied is unclear. It could be that the response changes reflect changes in the neuron’s modality-specific inputs, a change in its intrinsic operating characteristics, changes in inhibition within recurrent local circuits, changes in feedforward inhibition, or a mixture of these and other possibilities. The present study was designed to examine this issue by exploiting the multisensory properties of the SC “output” neurons of cats, which are a primary site of unisensory convergence (Fuentes-Santamaria et al., 2008, 2009; Meredith and Stein, 1985, 1986b; Meredith et al., 1992; Wallace et al., 1993). By examining whether the response changes induced by repetitions of a visual, auditory, or combined visual–auditory stimulus would “transfer” when the stimulus was “switched,” it was possible to determine whether modulatory response dynamics were applied before or after the transform that determines the neuron’s output.

## 2. Materials and methods

Two adult mongrel cats (1 female) were obtained from a USDA-licensed commercial animal breeding facility and will be referred to as F1 and F2. All procedures were conducted in accordance with the NIH Guide for the Care and Use of Laboratory Animals (National Institutes of Health Publication) and an approved Institutional Animal Care and Use Committee protocol at Wake Forest University School of Medicine, an Association for Assessment and Accreditation of Laboratory Animal Care-accredited institution. All efforts were made to minimize the number of animals used and each had been used in a previous study involving restricted unilateral visual cortex lesions. Animals was first screened to ensure that they were tractable and responded to visual and auditory stimuli in both hemifields at normal thresholds. However, only recordings in the SC of the intact hemisphere were conducted. Before beginning the test series, SC sensory responses were compared to those from controls to ensure the presence of normative responses to each of the stimuli and stimulus combinations to be used here (see Results).

### 2.1. Surgical procedures

Animals were anesthetized with a mixture of ketamine hydrochloride (20–30 mg/kg, IM) and acepromazine maleate (0.05–0.1 mg/kg, IM) and given antibiotics (5 mg/kg enrofloxacin, IM) and analgesics (0.005–0.01 mg/kg buprenorphine, IM). The surgical site was shaved, and the animal was intubated and placed in a stereotaxic head holder. The eyes were covered with a topical ophthalmic ointment, the site and body were draped, and surgical anesthesia was induced and maintained with isoflurane (induction: 3–5%, maintenance: 1–3.0%). Expiratory CO2, oxygen saturation, blood pressure, and heart rate were monitored using a vital signs monitor (VetSpecs VSM10), and body temperature was maintained with a heating pad. The skin and overlying muscle were reflected, and a craniotomy was performed to give access to the SCs on both sides of the brain. A stainless steel recording chamber was placed over the craniotomy and secured with stainless steel screws and dental acrylic (McHaffie and Stein, 1983). Upon completion of the surgery, the skin was sutured closed, and the implant was bathed with antibiotic ointment and lidocaine jelly. Anesthetic was discontinued and the animal was allowed to recover and was given the analgesics meloxicam (0.5 mg/kg, IM, sid for 3d) and buprenorphine (0.005–0.01 mg/kg, IM, bid), as well as the antibiotic cefazolin (20 mg/kg, IM, bid for 7d).

### 2.2. Recording procedures

Experimental recording sessions began more than a week after surgery. On each recording day, the animal was anesthetized with a mixture of ketamine hydrochloride (20–30 mg/kg, IM) and acepromazine maleate (0.05–0.1 mg/kg, IM), intubated with an endotracheal tube, and artificially respired. Respiratory rate and volume were adjusted to keep the end-tidal CO2 at 3.6–4.6%. The head was held by two horizontal posts that attached to the head holder without wounds or pressure points. An intravenous line was introduced in the hindlimb, and pancuronium bromide (0.1 mg/kg, IV) was used to prevent movement of the eyes or pinnae. Anesthesia, paralysis, and hydration were maintained using ketamine hydrochloride (2–10 mg/kg/h, IV) and pancuronium bromide (0.05 mg/kg/h, IV) in 5% dextrose in sterile saline (5 ml/h). Expiratory CO_2_, heart rate, and blood pressure were monitored continuously to assess depth of anesthesia (VetSpecs VSM10). The pupils were dilated with ophthalmic atropine sulfate (1%), and the eyes were fitted with contact lenses to prevent drying and to focus them on a tangent screen.

A single glass-coated tungsten electrode (tip diameter: 1–3 μm, impedance: 2–4 MΩ at 1 kHz) was lowered manually to the surface of the SC and advanced by a hydraulic microdrive to search for single neurons in the multisensory (i.e., deep) layers of the structure. Amplified neural signals were routed to an oscilloscope and audio monitor for on-line monitoring, to a window discriminator for the construction of rasters and peristimulus time histograms, and to a computer for storage. At the end of an experiment, drugs were discontinued, and the animal was allowed to recover stable respiration and coordinated locomotion before being returned to its housing unit.

### 2.3. Testing procedures

Visual search stimuli consisted of bars of light (5–10°) moved in various directions at different velocities. These stimuli were back projected onto a screen ~46 cm in front of the animal. Auditory stimuli (broadband noise, 20–20,000 Hz) were presented *via* a bank of stationary speakers (Panasonic model 4D02C0) arranged along an azimuth on a mobile hoop in 15° increments and 15 cm from the animal’s head. Auditory search stimuli consisted of broadband noise bursts (50 ms) from speakers or manual clicks, finger snaps, and hand claps. Once a neuron was isolated (signal/noise ratio = 3/1), and shown to respond to visual and auditory stimuli, its receptive fields (RFs) were mapped. Its visual RF was mapped by recording the site on the screen at which a bar of light, moved inward from all directions, initiated impulses. Auditory RFs were mapped *via* random activation of any of the speakers described above. Once a neuron’s RFs had been mapped, unisensory and multisensory response properties were examined quantitatively using a single visual stimulus and single speaker.

### 2.4. Neuron selection and test stimuli

A bar of light or noise burst (same as above) was presented within the overlapping RFs of an isolated multisensory neuron, with stimulus intensities customized to produce weak unisensory responses. However, no attempt was made to equilibrate stimulus effectiveness across modalities. They were presented individually or in a spatiotemporally congruent visual–auditory ensemble with a 25 ms visual-before-auditory delay (see Miller et al., 2015).

Each trial contained three repetitions (a “train”) of the same stimuli (visual V_1_, V_2_, V_3_; auditory: A_1_, A_2_, A_3_: or visual–auditory: VA_1_, VA_2_, VA_3_) followed by a fourth stimulus that was either the “same” (e.g., V → V) or was a “switch” to one of the other two stimuli (e.g., A → V). A response to the switch stimulus was therefore called the “switch response” and the response to the same stimulus was the “same response” (albeit its magnitude may have differed from those in the preceding train).

Preliminary studies identified a fixed and common presentation rate for which the visual or auditory train would reliably produce response changes but would not produce overlapping responses in either train. The response elicited by the first presentation would end before the next stimulus presentation began. A rate of 2 Hz (interstimulus interval = 500 ms) met these criteria, and produced attenuation in most of the auditory responses, but had variable effects on the visual responses. All neurons were tested with all nine pseudo-randomly interleaved stimulus conditions representing the pairing of the three possible trains (visual, auditory, visual–auditory) with a fourth stimulus having any of the three identities. These conditions were repeated for 20 rounds (inter-round interval = 2 s), resulting in each neuron being tested with a total of 180 trials.

### 2.5. Data analysis

Response magnitudes were calculated as the number of impulses elicited within 175 ms of stimulus onset, minus the expected number of spontaneously generated impulses (based on the firing rate in the 500 ms window preceding the stimulus train). Response latencies were identified using a three-step geometric method described by Rowland et al. (2007a,b,c). Spike density functions for each stimulus condition were calculated by convolving the trial-averaged impulse raster with a narrow symmetric kernel (N(0,8 ms)) and subtracting the spontaneous rate. These were used to estimate peak firing rates for responses. Cumulative impulse counts over time (“qsum,” see Rowland et al., 2007a,b,c) were calculated to provide a precise quantification of the temporal profile of the response without smoothing. Analyses were conducted neuron-by-neuron using time-averaged response magnitudes, trial-averaged spike density and qsum functions, and a stability analysis examining differences between responses at the beginning (first 5 trials) and end (last 5 trials) for each condition in each experiment.

#### 2.5.1. Metrics

The “baseline response” to each stimulus was calculated as the average response to its presentation whenever it was first in a train (i.e., V_1_, A_1_, VA_1_). Response changes (Δ) expected to be induced by a stimulus train on a response to the fourth stimulus by modulatory dynamics was calculated separately for each stimulus type. These changes were quantified as the proportionate difference between the response when the fourth stimulus had the same identity (“same response”) and the baseline response to that stimulus. For example, the visual proportionate change was calculated as:


(1) $$\Delta V=\frac{\left(V\to V\right)-V_{1}}{V_{1}}$$

For each switch response, a “transfer prediction” was generated for its expected value if the change induced by the stimulus train of one identity transferred across the switch to a stimulus of another identity. This was calculated by adding one to the proportionate ratio associated with the preceding stimulus train and multiplying it with the baseline for the switch response stimulus. For example, for switch response V → A, the transfer prediction G[V → A] was


(2) $$G\left[V\to A\right]=A_{1}\times \left(1+\Delta V\right)$$

For stimulus conditions involving multisensory responses, the transfer prediction was formed by a linear combination of the expected changes for the individual sensory channels. For example, when switching from a V train to a VA stimulus (switch response V → VA), the transfer prediction G[V → VA] was


(3) $$G\left[V\to VA\right]=VA_{1}\times \left(1+\frac{\left(\left(\left(V\to V\right)-V_{1}\right)+\left(\left(V\to A\right)-A_{1}\right)\right)}{V_{1}+A_{1}}\right)$$

The transfer prediction from a multisensory train to a modality-specific component stimulus (V or A) was structured in a similar way. For example, the transfer prediction G[VA → V] was


(4) $$G\left[VA\to V\right]=V_{1}\times \left(1+\frac{\left(\left(\left(V\to V\right)-V_{1}\right)+\left(\left(A\to V\right)-V_{1}\right)\right)}{2\times V_{1}}\right)$$

Multisensory response enhancement (ME_SF_) was calculated as the percent difference between the mean multisensory and most effective unisensory response calculated on a trial-by-trial basis *via* bootstrap, a metric referred to as “statistical facilitation” (SF) (Miller, 1982; Otto et al., 2013; Nava et al., 2014; Wang et al., 2020; Smyre et al., 2021; Bean et al., 2022).


(5) $$ME_{SF}=100\times \frac{\left(VA_{1}-SF\right)}{SF}$$

Briefly, the SF prediction was generated by repeatedly and randomly drawing (with replacement) responses on a single visual and a single auditory trial and calculating the maximum of the pair. This base procedure was repeated so that its iterations were equal to the number of multisensory trials presented to obtain a sample from which a single mean SF estimate was calculated. The base procedure was then repeated 10,000 times to generate the mean SF sampling distribution, which was used in statistical calculations described below. SF in Equation 5 is the mean of this distribution.

Because the data from each animal were very similar (see Supplementary Table 1), data were pooled across them. Unless otherwise specified, summary statistics are reported across neurons as the mean and standard error in the format mean ± standard error.

#### 2.5.2. Statistical significance

One sample and matched pairs t-tests were used to evaluate the mean of a sample relative to a referent value and the equality of means of matched samples, respectively. Welch’s t-tests were used to evaluate the equality of means of independent samples. For each neuron, baseline stimulus-driven responses were judged to be present when the mean response magnitude was significantly greater than 0. The visual, auditory, and visual–auditory stimulus trains were judged to induce significant changes whenever the mean magnitude of the same response (e.g., A → A) was significantly different from the mean magnitude of the associated baseline response (A_1_).

The value of p for multisensory enhancement was calculated as the proportion of the sampling distribution of the mean response magnitude predicted by SF that exceeded the mean multisensory response magnitude (see Wang et al., 2020). Multisensory neurons (the only neurons studied here) were sorted into the following exclusive categories: neurons with significant enhancement that were responsive to both sensory modalities (“overt multisensory neurons”) and neurons without significant enhancement but responsive to both modalities (“non-integrating multisensory neurons”). Linear regression was used to evaluate predictive relationships. The standard for significance throughout was alpha = 0.05.

## 3. Results

**Figure 1:**
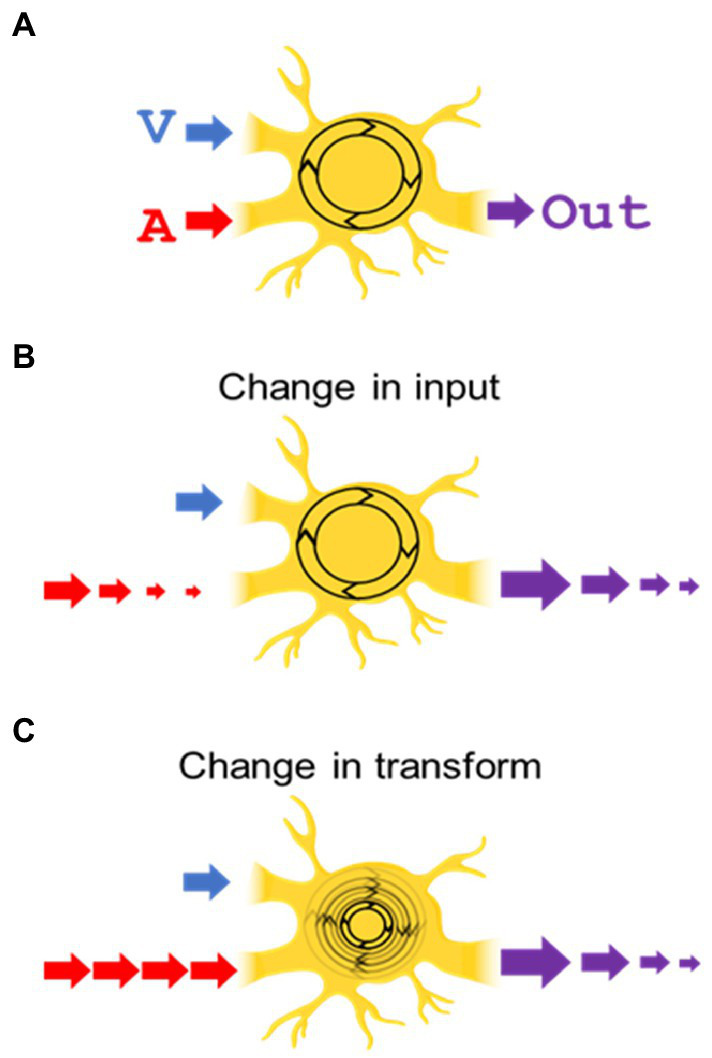
Do the modulatory dynamics based on experience alter a neuron’s unisensory inputs, or its transform? **(A)** Depicted is a neuron with visual (“V”) and auditory (“A”) inputs being integrated by its inherent transform (shown as a rotating circle) and producing a multisensory output (“Out”). **(B)** In the “change in input” hypothesis, repetition of the (auditory in this illustration) stimulus decreases its input to the transform, leading to a diminished multisensory output. **(C)** In the “change in transform” hypothesis, the input strength is unchanged, but the neuron’s transform is altered, decreasing its multisensory output.

One issue explored here was where modulatory dynamics induced by trains of the same stimulus are applied. The alternatives are illustrated in Figure 1. One possibility is that modulatory dynamics are generated and applied separately within the neuron’s individual sensory input channels, i.e., in the visual and auditory channels “upstream” of the target neuron. An alternative is that these dynamics modify the general operation (“transform”) of the SC neuron. A second issue is whether the dynamics are dependent on the output of the neuron, which varies depending on whether the stimulus is visual, auditory, or visual–auditory.

### 3.1. Baseline response properties

**Figure 2:**
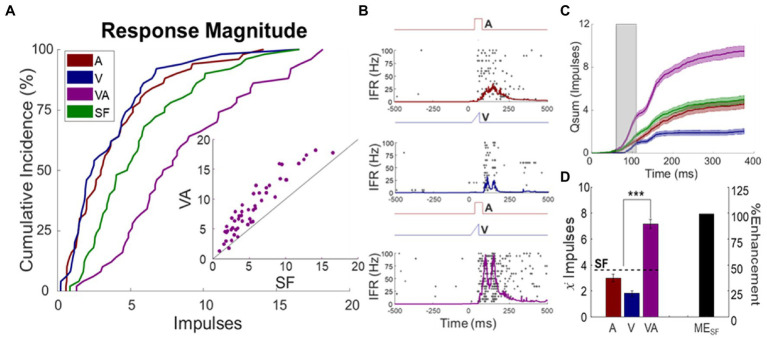
Sensory responses of multisensory SC neurons. **(A)** Cumulative distributions of the visual (V), auditory (A), and visual–auditory (VA) baseline responses in the population, along with the predictions based on statistical facilitation (SF). Inset scatter plot (each circle represents one neuron) shows that the mean VA response was mostly enhanced over the SF prediction on a neuron-by-neuron basis. **(B)** Multisensory enhancement in an exemplar neuron. Impulse rasters and spike density functions showed the typical pattern of response enhancement (each dot = 1 impulse, each row = 1 trial, trials are ordered from bottom-to-top). Labelled electronic traces above each raster show the stimulus time course. **(C)** The plots of cumulative impulse counts over time for each response illustrate that multisensory enhancement was most robust in the initial window of input overlap (shaded). **(D)** The bar graph summarizes the response magnitude for each condition. Note that the mean multisensory enhancement exceeded statistical facilitation.

The sample of multisensory neurons studied here (*F*_1_ = 32; *F*_2_ = 18) had response properties that were similar across animals and with those reported previously (Stein et al., 1973a,b; Meredith and Stein, 1983, 1986b; Stanford et al., 2005; Yu et al., 2009; Miller et al., 2015). Thus, the data were pooled. The mean spontaneous firing rate was 1.9 ± 0.38 impulses/s. All neurons responded overtly to the auditory stimulus (3.9 ± 0.46 impulses), to the visual stimulus (3.6 ± 0.44 impulses), and to the visual–auditory stimulus combination (8.6 ± 0.64 impulses). Auditory responses typically exhibited shorter latencies (16 ± 1.1 ms), with peak firing rates of 67 ± 7.4 impulses/s and mean durations of 109 ± 6.6 ms. Visual responses had longer latencies (65 ± 3.6 ms) with peak firing rates of 55 ± 5.5 impulses/s and durations of 122 ± 6.3 ms. Visual–auditory responses had latencies of 43 ± 1.3 ms, with peak firing rates of 114 ± 6.5 impulses/s and durations of 130 ± 4.4 ms. Ninety-four percent of neurons exhibited significant multisensory enhancement (ME_SF_: 67 ± 6.6%, see exemplar Figure 2). Ninety four percent of them were categorized as overt multisensory neurons and 6% as non-integrating multisensory neurons. The incidence of overt multisensory neurons was higher than reported in previous studies (see (Meredith and Stein, 1983, 1986b; Meredith et al., 1987; Stein and Meredith, 1993; Miller et al., 2017; Bach et al., 2018), reflecting a bias in the present study toward neurons most appropriate to address the questions posed here.

Of the integrating neurons, the largest enhancements were observed at the beginning of the window in which the inputs were most likely to overlap (Rowland et al., 2007a; Rowland and Stein, 2008; Miller et al., 2015, 2017). Baseline response properties of these neurons proved to be stable during experimentation; there were no significant changes in response magnitudes during the experiment for any of the stimulus conditions (auditory: *p* = 0.4115, visual: *p* = 0.9119, visual–auditory: *p* = 0.6942).

### 3.2. Responses to stimulus trains

The effect of modulatory dynamics on multisensory SC neurons was explored using modality-specific and cross-modal stimuli repeated in 2 Hz trains.

#### 3.2.1. Auditory responses

**Figure 3:**
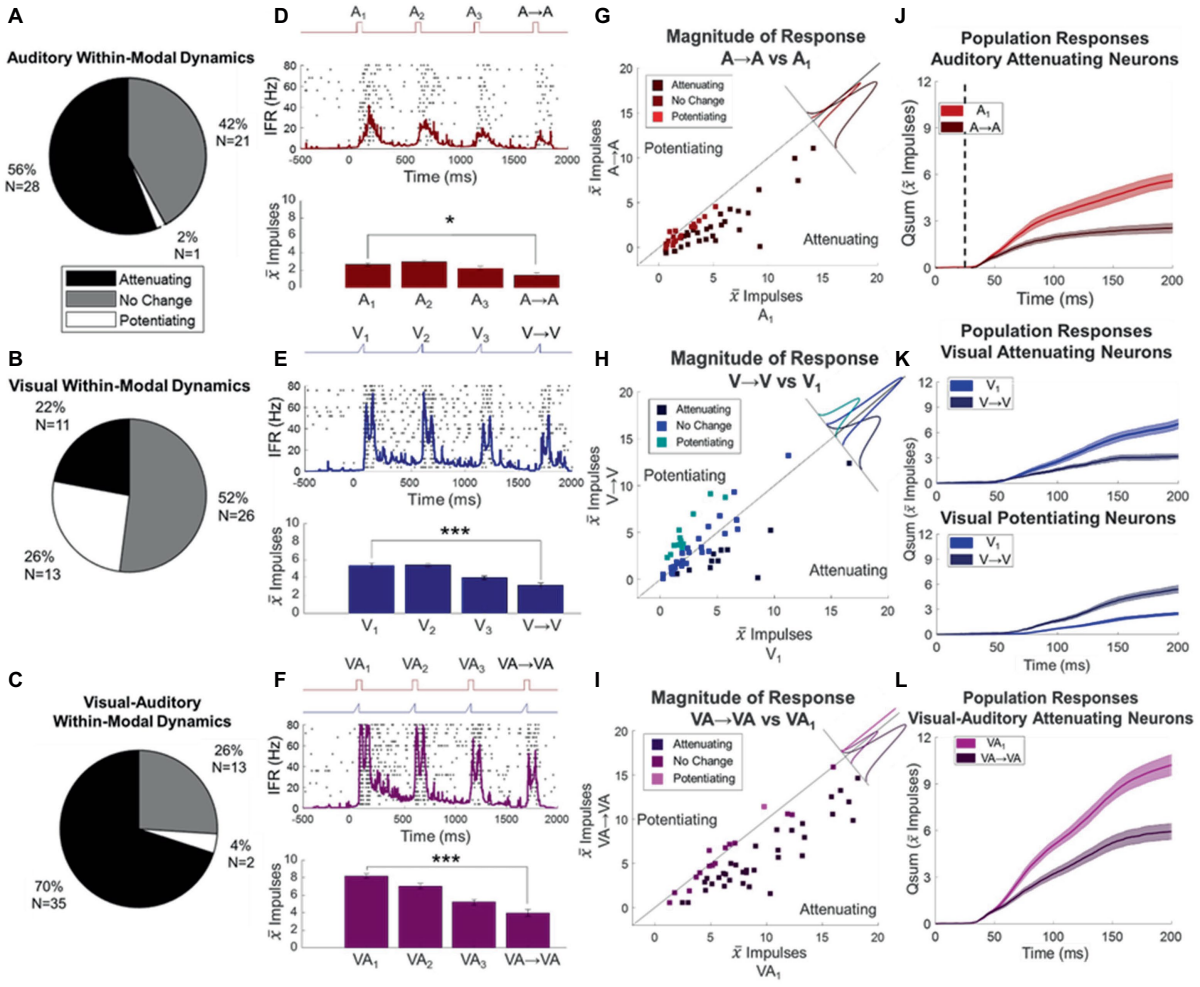
Response changes elicited by stimulus trains. **(A,B,C)** Pie charts describe the proportion of neurons whose responses attenuated or potentiated to the auditory **(A)**, visual **(B)**, or visual–auditory **(C)** stimulus trains. **(D,E,F)** Shown are the spike density functions of an exemplar neuron’s response to the auditory **(D)**, visual **(E)**, and visual–auditory **(F)** stimulus trains overlaid on their respective impulse rasters. Conventions are the same as in **(B)**. Bar graphs below show progressive attenuation in mean response magnitudes in both cases. *p < 0.05, ***p < 0.001. **(G–I)** Shown are scatter plots for each stimulus type comparing baseline (V_1_, A_1_) to the “same response” (V- > V, A- > A) for the auditory **(G)**, visual **(H)**, and visual–auditory **(I)** stimulus types. Color indicates whether the responses in the train showed significant attenuation (darkest) or potentiation (lightest). Solid lines indicate the line of unity (no change), perpendicular insets show the ¯marginal distribution of deviations from the line of unity grouped by direction of the change observed. Points above the line of unity are potentiated and below are attenuated. **(J−L)** Plotted here are the cumulative impulse counts over time for the population of auditory attenuating **(J)**, visual attenuating (**K**, top), visual potentiating (**K**, bottom), and visual–auditory attenuating **(L)** neurons. Dashed vertical line indicates auditory stimulus onset. They show that the effect of the modulatory dynamics was early in the response.

On average across the entire sample of neurons (*N* = 50), the unisensory auditory train induced significant attenuation of response magnitude (proportionate change ΔA = −0.52 ± 0.06, *p* < 0.0001, see exemplar Figure 3D). This response attenuation was significant in 56% (28/50) of the individual neurons within the sample, and only one showed significant response potentiation (Figures 3A,G). The average proportionate change (ΔA) in neurons showing significant attenuation was −0.70 ± 0.08. The timing with which the modulatory dynamics affected the probe response was examined by subtracting the temporal profiles (qsums) of the probe (i.e., A → A) and baseline (i.e., A_1_) responses after aligning the stimulus onsets. These temporal profiles diverged very early in the response (22 ± 5.9 ms, stimulus onset at 25 ms in Figure 3J).

#### 3.2.2. Visual responses

In contrast, the visual train produced no significant changes in response magnitude at the population level (proportionate change ΔV = 0.26 ± 0.15, *p* = 0.9094, see exemplar Figure 3) at these iterative rates, although there was some diversity at the individual neuron level. A slight majority (52% = 26/50) did not show significant changes; however, responses were significantly attenuated in 22% (11/50) and potentiated in 26% (13/50) of them (Figures 3B,H). In the neurons showing response attenuation, the proportionate change (ΔV) was −0.55 ± 0.06. And, as noted above for the auditory modality, the effect of the modulatory dynamics became apparent early in the response, with the temporal profile of the V → V response diverging from baseline 31 ± 10 ms after response onset. In the neurons showing response potentiation, the proportionate change in response magnitude was ΔV = 1.4 ± 0.19. And again, the effect of the modulatory dynamics was seen early in the response window (divergence of V → V and baseline responses occurred 25 ± 11 ms after response onset, Figure 3K).

#### 3.2.3. Multisensory responses

Repeated presentation of visual–auditory stimuli in a 2 Hz stimulus train induced complex response patterns. The mean response change (ΔVA) across the population (N = 50) was a significant decrement (−0.32 ± 0.04, p < 0.0001). They induced response attenuation in 70% of the sample (*N* = 35) and, in those in which that attenuation was statistically significant, the decrement averaged −0.44 ± 0.03. In several cases (26%, *N* = 14, Figures 3C,I), no significant changes were noted and in only one case was potentiation elicited. The temporal profiles of the VA → VA response also changed, diverging early from the baseline comparator responses. They did so (divergence of VA → VA) soon after response onset (27 ± 5.2 ms, Figure 3L).

There was no correlation between the response changes produced in any given neuron by the visual and auditory trains (Spearman’s, *p* = 0.95). The presence and implications of this modality-specific distinction between the responses of the same neuron to its visual and auditory inputs became more apparent in other tests, as described below.

### 3.3. Modulatory dynamics did not transfer between sensory modalities

**Figure 4:**
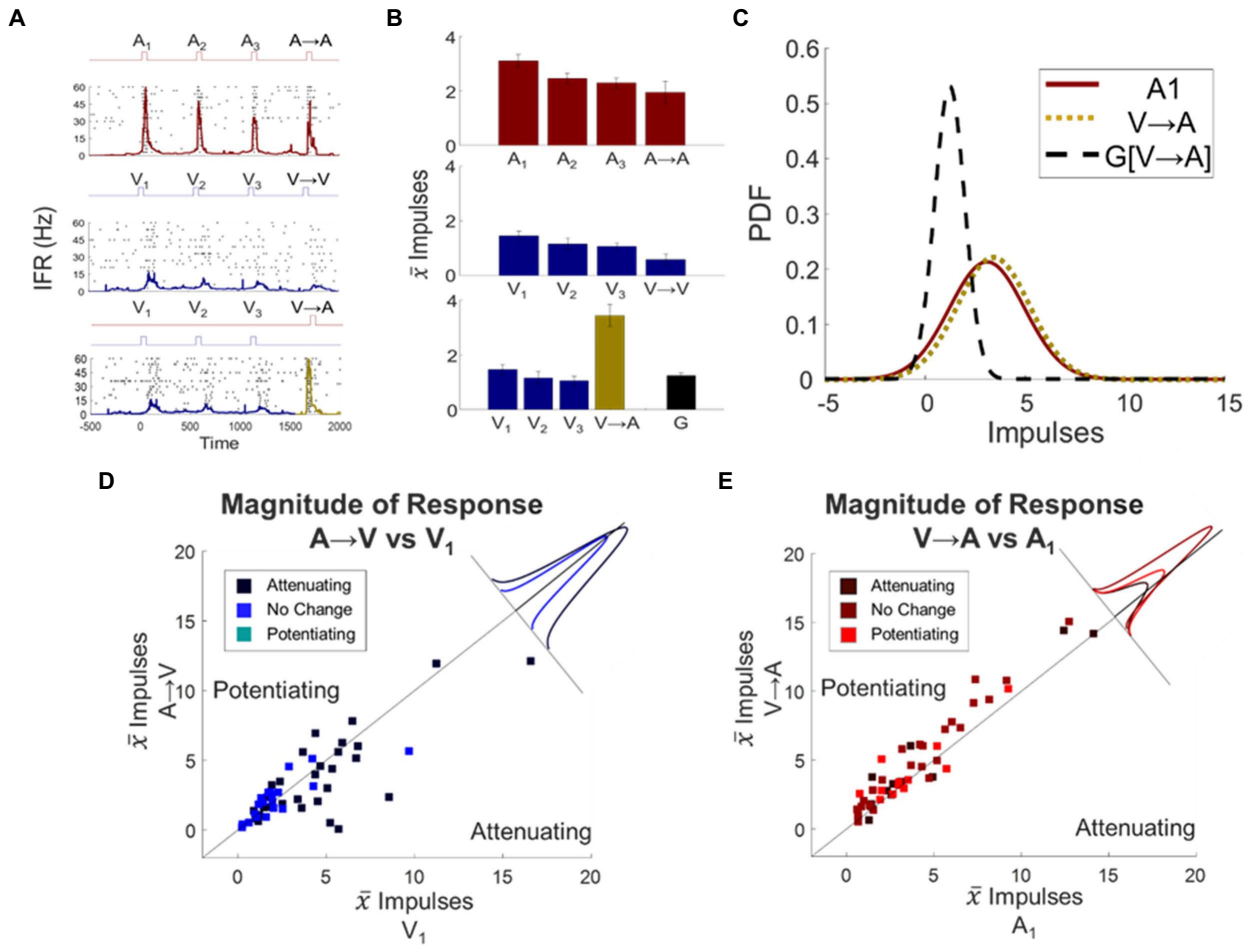
Unisensory response changes induced by stimulus trains did not transfer across modalities: they were channel specific. **(A)** Spike density functions overlaid on response rasters illustrate an exemplar neuron’s responses to an auditory stimulus train, a visual stimulus train, and a visual train followed by the switch to an auditory stimulus. Conventions are the same as in preceding figures. **(B)** Shown are mean response magnitudes for each of the responses illustrated in **(A)**, along with transfer prediction G indicating the expected magnitude of the V → A response if the visual train dynamic transferred to the auditory modality. Note that V → A (gold bar) was far greater than predicted. **(C)** Illustrated here are the probability distribution functions (PDF) for baseline A_1_, switch response V → A, and the transfer prediction G[V → A]. Note that the switch response closely resembled the baseline A_1_ response, indicating the absence of transfer. **(D)** Shown here is a scatter plot of the mean responses of each neuron to baseline V_1_ response versus the A → V switch response. They are grouped by color according to whether the auditory train produced potentiation, attenuation, or no change. The inset shows the distribution of deviations from the line of unity for each group. Conventions are the same as Figure 3. **(E)** The same conventions are repeated for the relationship of baseline A_1_ to V → A. In neither the A → V nor the V → A switch condition did the dynamic induced by the preceding stimulus train transfer across modalities.

The distinction noted above suggested that a neuron’s modulatory dynamics were specific to its individual input channels. If so, the dynamic induced by a stimulus train in one modality (attenuating or potentiating) would fail to “transfer” to the response when the stimulus was switched to the other modality. Alternatively, if the modulatory dynamics have a general effect on the target neuron, the response changes induced by a train of stimuli in one modality would transfer to those elicited by the other (see Figures 4A–C). To examine this issue, the fourth and final stimulus in a train was switched, either from A to V (eliciting “switch response” A → V) or from V to A (eliciting “switch response” V → A).

In neurons in which the auditory stimulus train produced significant attenuation (*N* = 28), the A → V switch responses were affected: they were significantly different from the visual baseline response (Δ = −0.85 ± 0.42 impulses, *p* = 0.0486, Figure 4D). However, on an individual neuron basis, the majority (61%; 17/28) were not consistent with this and instead showed a lack of an effect. Only a minority of them showed an increase (10%; 3/28) or a decrease (29%; 8/28) in response magnitude, and even among those neurons the switch response A → V was always closer to the baseline than to the transfer prediction. In short, the modulatory dynamics of the auditory response did not transfer to the visual response.

A similar finding was observed in the V → A switch condition. In the population in which the visual train predicted attenuation, the V → A switch responses were not significantly different from baseline (Δ = 0.61 ± 0.35 impulses, *p* = 0.1132, Figure 4E). At the individual neuron level, there was no significant change from the auditory baseline response in 73% (*N* = 8) of the neurons, there was potentiation (the opposite of the transfer effect) in 18% of them (*N* = 2), and only one neuron showed significant attenuation (yet its response was closer to the baseline than to the transfer prediction). The results were similar for the population in which the visual train predicted potentiation (Δ = 0.77 ± 0.37 impulses, *p* = 0.0602). In this group, 77% (*N* = 10) of the neurons showed no significant change. The switch response potentiated in only three of them (23%) and in two out of the three cases the switch response was closer to the baseline response than to the transfer prediction.

These observations strongly suggest that the modulatory dynamics induced by repeated modality-specific stimuli are applied independently to the multisensory neuron’s unisensory input channels: they do not transfer across them. The few marginal cross-sensory effects that were observed were often of opposite sign, and overall uncorrelated with the transfer prediction (Spearman’s, *p* = 0.2463). These were used as regressors alongside others in the following analyses involving the multisensory responses of these neurons.

### 3.4. Modulatory dynamics transfer from unisensory to multisensory responses

**Figure 5:**
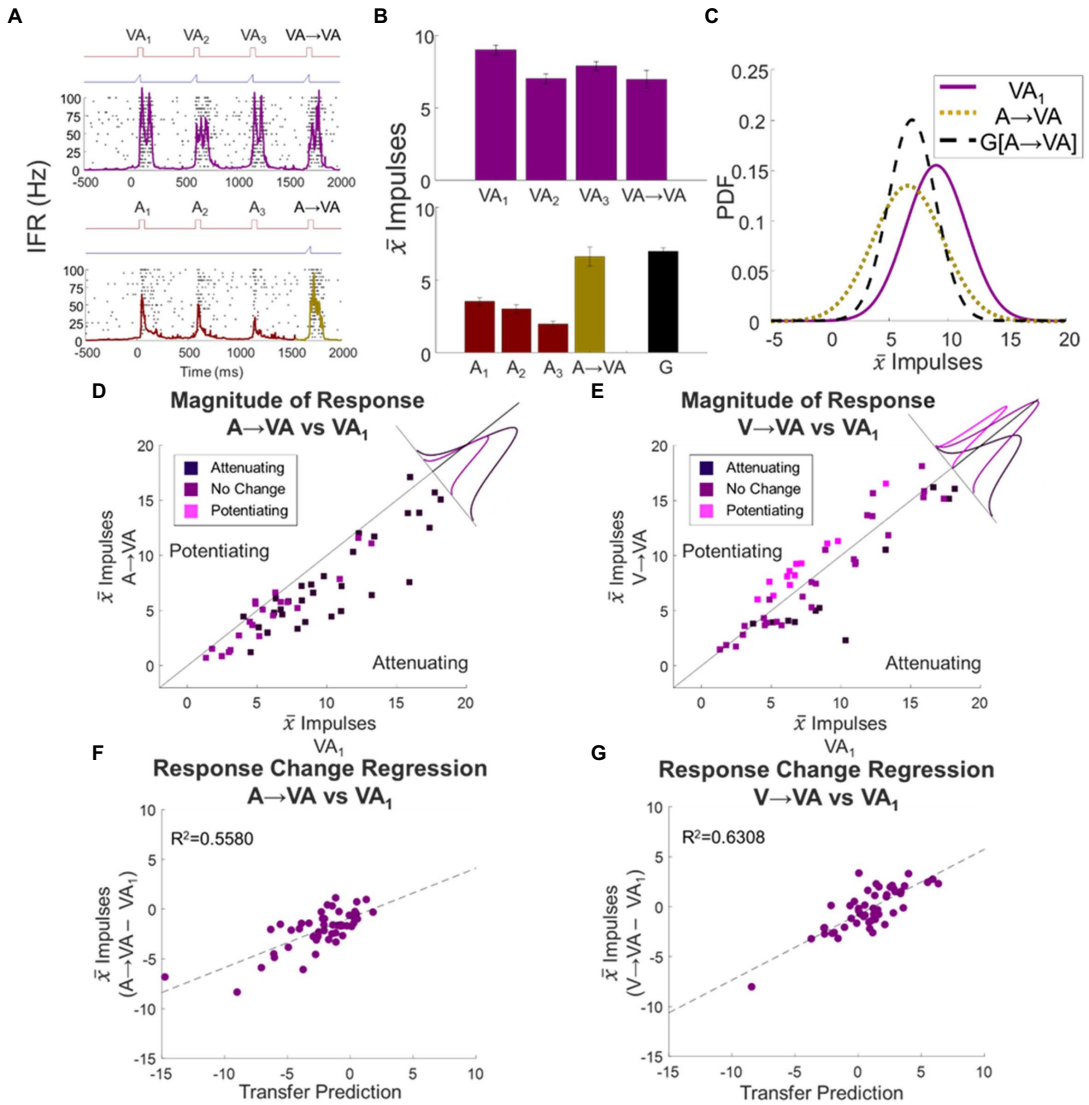
Unisensory response changes transferred to multisensory responses regardless of modality and/or direction of change. **(A–C)** The evaluation of transfer for a single exemplar neuron between the A and VA conditions is illustrated here. Conventions are the same as Figures 4A–C. **(D, E)** Shown are scatter plots comparing mean baseline VA_1_ to the switch responses A → VA **(D)** and V → VA **(E)**. Insets show the distribution of deviations from the line of unity for each group. When the response to the preceding auditory stimuli was attenuated, the response to the switch stimulus (A → VA) was also attenuated. Similarly, following a switch from a visual train, the multisensory switch response was consistent with the modulating dynamics expected from the preceding visual stimulus train, regardless of whether it potentiated (pink) or attenuated (dark purple). **(F,G)** The deviations of the multisensory response from baseline were well-predicted by the transfer prediction when switching to a multisensory condition after either an auditory **(F)** or visual **(G)** stimulus train. Dashed lines indicate linear regression fits.

The above results revealed that modulatory dynamics operate on the multisensory neuron’s individual sensory input channels independently, with some unrelated and marginal cross-sensory effects. This suggests that the dynamics induced by a train of modality-specific (i.e., visual, or auditory) stimuli would transfer when the switch was from a modality-specific train to a cross-modal (i.e., visual–auditory) probe. Nevertheless, the strong alternative is that the cross-modal condition would be processed as unique, just as the separate modalities are processed as unique. If so, the modulatory dynamics would not transfer, and the multisensory response would not deviate from baseline (see Figures 5A–C). These hypotheses were first tested by examining responses to the visual–auditory probe stimulus that followed auditory or visual stimulus trains.

In the population in which the auditory stimulus train produced significant response attenuation (*N* = 28), the switch response to the cross-modal stimulus (A → VA) was also significantly attenuated (Δ = −2.8 ± 0.08 impulses, *p* < 0.0001). This difference was statistically significant in 64% (*N* = 18) of the constituent neurons, and most (80%) of the remainder trended in the same direction (Figure 5D). This supported the principle that the modulatory dynamic generated by the modality-specific stimulus train transferred to the multisensory probe response. To determine if the effects on the multisensory response were quantitatively predictable from the effects of the stimulus train on its presumptive component responses, the attenuation of the switch response (A → VA – VA_1_) was regressed against the sum of those constituent responses when examined individually (A → A – A_1_) + (A → V – V_1_). This regression was highly significant (slope = 0.5002, intercept = −0.8805, *R*^2^ = 0.5580, *F* statistic = 60.59, *p* < 0.0001; Figure 5F). Thus, not only was the cross-modal stimulus condition not processed as unique from the preceding auditory stimulus condition, but the response it elicited was consistent with the stimulus train effects being applied independently to each of the neuron’s unisensory input channels.

The same result was seen in switches from a visual train to the cross-modal probe stimulus. In cases in which the visual train induced significant attenuation, V → VA responses were also significantly attenuated (Δ = −2.6 ± 0.64 impulses, *p* = 0.003). This trend was present in a significant number of neurons in this group (*N* = 10/11) and statistically significant in 82% (*N* = 9) of them. For samples in which the visual train induced significant potentiation, V → VA responses were also significantly potentiated (Δ = 2.0 ± 0.17 impulses, *p* < 0.0001). Again, almost all samples were consistent with this trend, which was statistically significant in 77% (*N* = 10) of them (Figure 5E). And again, there was a quantitative relationship between the change in the multisensory switch response from baseline (V → VA – VA_1_) and the sum of changes induced by the visual train on the unisensory component responses (slope = 0.6556, intercept = −0.7978, *R*^2^ = 0.6308, F statistic = 82.01, *p* < 0.0001; Figure 5G). This result underscored the independence of the modulatory dynamics on the input channels, as it was apparent even in the presence of multiple modality-specific stimuli.

### 3.5. Modulatory dynamics transfer from multisensory to unisensory responses

**Figure 6:**
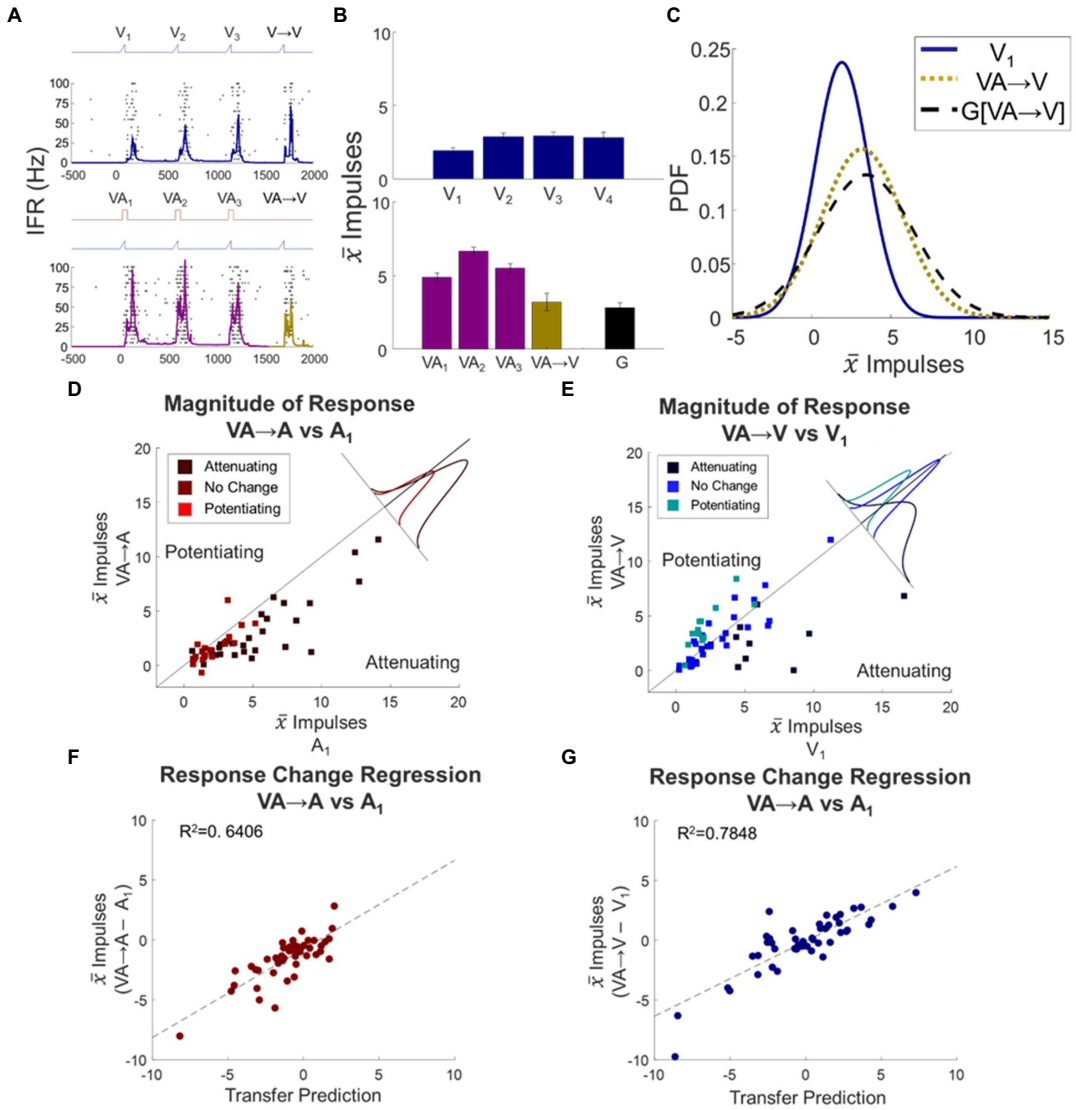
Multisensory response changes did transfer to their unisensory component responses. Conventions are the same as Figure 4, but now what is being compared is the transfer from VA stimulus trains to switches in which either a single auditory or single visual probe stimulus is presented. **(A–C)** Conventions are the same as Figures 4, 5, here illustrating that the visual dynamic expected from a VA train does transfer across to the visual switch response. **(D,E)** Scatter plots compare the mean baseline and switch responses to evaluate the transfer of the modulatory dynamics induced by VA stimulus trains to auditory **(D)** or visual **(E)** probe responses, showing that the switch responses deviate from baseline in predictable ways. Conventions the same as Figure 4. **(F,G)** Scatter plots compare deviations from baseline for the VA → A **(F)** and VA → V **(G)** switch responses and their respective transfer predictions. They show good matches in each case. Conventions are the same as Figure 5.

Because the preceding stimulus trains in the analyses above involved only a single modality, they did not address the derivation of those modulatory dynamics. The most obvious possibility is that they are derived independently from each modality, just as they are independently applied. In this case, the dynamics induced by the component stimuli in a visual–auditory train should transfer across a switch to a component modality. A strong alternative, however, is that modulatory dynamics are derived from (or depend on) the (multisensory) output of the neuron. If so, they would fail to transfer across such a switch (see Figures 6A–C).

When examining these possibilities by switching from a visual–auditory train to an auditory stimulus, the VA → A switch response was significantly attenuated relative to baseline (Δ = −1.5 ± 0.25 impulses, *p* < 0.0001, Figure 6D). To evaluate the relationship between the switch response and the transfer predictions quantitatively, the (VA → A – A_1_) change was regressed against the sum of the effects of the individual auditory and visual stimulus trains: (A → A – A_1_) + (V → A – A_1_). The regression was highly significant (slope = 0.7397, intercept = −0.7480, *R*^2^ = 0.6406, *F* statistic = 85.55, *p* < 0.0001; Figure 6F, inset), suggesting that the dynamics were not derived from multisensory outputs but from a linear combination of the changes within the auditory and visual input channels.

The results observed when the visual–auditory train was switched to a visual stimulus were similar, although the patterns predicted were more complex because the effects of the modulatory dynamics on the visual modality were more variable. In the entire cohort, there was no significant difference from baseline in the VA → V response (Δ = −0.33 ± 0.37 impulses, *p* = 0.3714, Figure 6E). Nonetheless, here too there was a highly significant quantitative relationship between the (VA → V – V_1_) change and the sum of the effects of the auditory and visual trains: (A → V – V_1_) + (V → V – V_1_) (slope = 0.6273, intercept = −0.0712, R^2^ = 0.7848, F statistic = 175.1, p < 0.0001; Figure 6G, inset). This result again revealed that the visual–auditory stimulus induced modality-specific dynamics that were independently sourced from and applied to the individual sensory channels.

## 4. Discussion

The predictability of an external event (e.g., a stimulus) changes sensory responses throughout the nervous system (Berns et al., 2001; McMahon and Olson, 2007; Egner et al., 2010; Esterman and Yantis, 2010; Meyer and Olson, 2011; Kok et al., 2012; de Gardelle et al., 2013). One of the best-known examples of this is the decrease in a neuron’s response vigor to rapid trains of an identical innocuous stimulus, an effect often referred to as “habituation” or “attenuation” (the latter term is preferred here due to semantic overloading of the former). This effect, which has been repeatedly described in neurons of the SC, causes a stimulus to lose access to the circuitry of this sensorimotor structure and thereby lose its ability to initiate orientation responses (Fecteau and Munoz, 2005; Reches and Gutfreund, 2008; Boehnke et al., 2011; Netser et al., 2011; Dutta and Gutfreund, 2014; Bean et al., 2021). Changing the features of that stimulus, or the context in which it appears (i.e., violating predictability), restores responsiveness and access to this circuit and the orientation responses it induces (“dishabituation”) (Sokolov, 1963; Hoffman and Stitt, 1969; Reches and Gutfreund, 2008; Boehnke et al., 2011). Repeating stimuli at different iterative rates can also induce the opposite change: a strengthening of the response vigor (“potentiation”), which often represents the sensitization of the sensorimotor apparatus to a weakly-effective stimulus (Hughes et al., 1956; Zhang et al., 2000; Perrault et al., 2011; Kordecka et al., 2020). Both effects were observed here.

The simplicity of these effects belies the complexity of the underlying neural mechanisms. How is a neuron’s response modulated by the “predictability” of an event? As noted earlier, there are several plausible possibilities. Predictability could be calculated based on whether the neuron’s input is changing or whether its output is changing. Similarly, the effects of predictability could be applied (*via* modulatory dynamics) on the neuron’s inputs, affecting them in a specific way, or to the neuron itself (e.g., its membrane properties, or local circuit dynamics), thereby affecting its general excitability. The multisensory nature of SC neurons makes them ideal for addressing this question because their inputs and outputs are distinct, and the former can be independently manipulated (Huerta and Harting, 1982; Meredith and Stein, 1985; Sparks, 1986; Meredith et al., 1992; Wallace et al., 1993; Fuentes-Santamaria et al., 2009; Stein et al., 2009). To address this question, we determined whether the modulatory dynamics induced by repeated stimuli would transfer, or not, when the identity of the stimulus was switched to a different modality, or when a stimulus of another modality was added to or subtracted from that stimulus train.

As noted above, the effects of modulatory dynamics induced by the stimulus train transferred when a probe stimulus had the same identity (e.g., auditory), but not when its modality changed (e.g., a switch from auditory to visual), regardless of whether the effects of the modulatory dynamic were attenuating or potentiating. This result might be expected based on the assumption that a switch from one sensory modality to the other represents a substantial change in stimulus features, even though the visual and auditory stimuli originated from the same location. The more illuminating and less expected result was that these dynamics transferred between unisensory and multisensory conditions. It was less expected because a visual–auditory probe stimulus following a visual or auditory stimulus train also represents a significant change in stimulus features, as does the single visual or auditory probe stimulus presented after a visual–auditory train of stimuli. These are stimulus changes that prior literature suggests would cause the modulatory dynamic to fail to transfer (e.g., dishabituation should occur).

However, the transfer that was observed here can be understood as indicating that modulatory dynamics are generated from, and independently applied to, the neuron’s unisensory inputs; that is, prior to the (multisensory) transform by which a neuron’s inputs are converted to its output. If these modulatory dynamics were generated from (i.e., based on) the neuron’s output, which is different in multisensory vs. unisensory conditions, then they would have failed to transfer when switching between modality-specific trains and cross-modal probe stimuli and *vice-versa*. If the dynamics were applied to the neuron generally (e.g., raising or lowering its excitability), and not to its unisensory inputs, then they should have transferred in the switch between visual trains and auditory probe stimuli, and vice versa.

This does not mean that no cross-modal influences were observed. Indeed, there were effects of auditory trains on visual probe responses, and vice versa, and these were useful regressors in predicting the neuron’s responses to switches between unisensory and multisensory conditions. However, these cross-modal effects were not predictable from the modulatory dynamics influencing changes in the preceding train. One possibility is that they reflected (unpredictable) persistent changes in the neuron’s membrane properties or local circuit induced by stimulus trains (e.g., “rebound” effects, see (Grenier et al., 1998; Zheng and Raman, 2009; Wang et al., 2016). An alternative is that these changes may represent a larger category of cross-modal interactions only partially probed here, that would be more fully illuminated by experimentation with a broader set of stimulation frequencies and stimulus asynchrony parameters (Cuppini et al., 2020).

There is significant value in applying prediction-based modulatory dynamics to multisensory neurons in a modality-specific manner. Perhaps most apparent is the ability to diminish the impact of predictable and “meaningless” modality-specific cues so that they do not compete with other, potentially important, cues for access to the detection/orientation circuitry of the SC. This works in a similar but opposite way for potentiating dynamics. However, these goals could not be realized if modulatory dynamics transferred between sensory channels in complex environments in which there is often spatial overlap between unrelated and independent events. The modality-specific application of modulatory dynamics also preserves the unique perceptual experience associated with each sense and the ability for each sense to operate independently when needed (Duncan et al., 1997; Colonius and Diederich, 2004; Conway and Christiansen, 2006; Seitz et al., 2007; Bean et al., 2021).

It is important to recognize that the results obtained here are specific to neurons (like those of the SC) that are primary sites of multisensory convergence, and that these neurons are generous in their projections. The widespread outputs from SC neurons generally make it likely that the factors determining their responses may strongly influence those of many neurons elsewhere in the brain; for example, in the posterior thalamus, which then projects to cortical targets (Harting et al., 1973; Kawamura, 1974; Holstege and Collewijn, 1982; Abramson and Chalupa, 1988; Comoli et al., 2003). Whether other sensory projections significantly alter the impact of these direct and indirect SC targets, and whether other multisensory neurons in these or other brain regions have adopted different sets of modulatory dynamics to establish their predictive coding capabilities (Talsma, 2015; Shi and Burr, 2016), remains unknown.

So too are the circuit components responsible for implementing the specific modulatory dynamics observed here. SC neurons are embedded in local circuits and receive inputs from a host of subcortical and cortical sources, many of which are sensitive to experience and whose neurons have remarkable plasticity (Zhang et al., 2001; May, 2006; Wallace et al., 2006; Lomber et al., 2010; Maya-Vetencourt and Origlia, 2012; Li et al., 2017). One possibility is that the effects represent modulation of the tectopetal inputs from the anterior ectosylvian sulcus and rostral lateral suprasylvian sulcus. These inputs are known to be critical for the multisensory integrative functions of SC neurons and for their experience-based plasticity (Jiang et al., 2001, 2006; Stein et al., 2002; Alvarado et al., 2009; Yu et al., 2014). These afferents, operating independently or collectively with one another, and/or in tandem with other structures (e.g., zona incerta Edwards et al., 1979; Roger and Cadusseau, 1985; Ficalora and Mize, 1989; May et al., 1997) could support the complex computations underlying the modulation of neuronal responses by stimulus predictability. Also possible is that these rapid effects could represent changes within short-latency inputs from subcortical sources projecting to the intermediate and deep layers of the SC (e.g., see Edwards et al., 1979; Grantyn et al., 1984; Huffman and Henson, 1990; Behan and Appell, 1992).

## Data availability statement

The raw data supporting the conclusions of this article will be made available by the authors, without undue reservation.

## Ethics statement

The animal study was reviewed and approved by Institutional Animal Care and Use Committee protocol at Wake Forest University School of Medicine, an Association for Assessment and Accreditation of Laboratory Animal Care-accredited institution.

## Author contributions

SS, NB, BS, and BR designed research. SS and NB performed research. SS and NB analyzed data. SS, NB, BS, and BR wrote the paper. All authors contributed to the article and approved the submitted version.

## Funding

The Tab Williams Foundation, the National Institutes of Health (Grants RO1 EY031532 and T32 NS073553).

## Conflict of interest

The authors declare that the research was conducted in the absence of any commercial or financial relationships that could be construed as a potential conflict of interest.

## Publisher’s note

All claims expressed in this article are solely those of the authors and do not necessarily represent those of their affiliated organizations, or those of the publisher, the editors and the reviewers. Any product that may be evaluated in this article, or claim that may be made by its manufacturer, is not guaranteed or endorsed by the publisher.
