# Supplementary material for: Predictability alters multisensory responses by modulating unisensory inputs
Source: Front Neurosci. 2023 Mar 29;17:1150168. doi: 10.3389/fnins.2023.1150168 (PMC10090419; doi:10.3389/fnins.2023.1150168)
Supplement: Supplementary file 1 [file Table_1.DOCX]

| Supplementary Table 1 | | | | |
| --- | --- | --- | --- | --- |
|  | | Mean ± Standard Error | | |
|  |  | Combined  N=50 | F_1_  N=32 | F_2_  N=18 |
| Auditory | Response | 3.9±0.46 | 3.9±0.58 | 4.0±0.79 |
|  | Latency | 16±1.1 | 15±1.5 | 16±1.6 |
|  | Peak Firing Rate | 67±7.4 | 62±9.2 | 77±12 |
|  | Duration | 109±6.6 | 122±8.6 | 85±7.8 |
| Visual | Response | 3.6±0.44 | 2.7±0.38 | 5.2±0.92 |
|  | Latency | 65±3.6 | 68±2.7 | 59±8.7 |
|  | Peak Firing Rate | 55±5.5 | 42±6.8 | 77±9.1 |
|  | Duration | 122±6.3 | 122±7.8 | 122±11 |
| Multisensory | Response | 8.6±0.64 | 8.0±0.75 | 9.6±1.2 |
|  | Latency | 43±1.3 | 42±1.6 | 45±2.2 |
|  | Peak Firing Rate | 114±6.5 | 107±8.2 | 128±11 |
|  | Duration | 130±4.4 | 138±5.3 | 116±6.4 |
| Enhancement | ME_SF_ | 67±6.6% | 79±7.8% | 46±11% |
| A→A | Δ A ↓ | -0.70 ± 0.08 | -0.70 ± 0.10 | -0.69 ± 0.13 |
|  | Latency of Attenuation | 22±5.9 | 25±15 | 15±7.3 |
| V→V | Δ V ↓ | -0.55±0.06 | -0.60±0.13 | -0.52±0.06 |
|  | Latency of Attenuation | 31±10 | 30±22 | 32±12 |
|  | Δ V ↑ | 1.4 ± 0.19 | 1.4 ± 0.23 | 1.2 ± 0.17 |
|  | Latency of Potentiation | 25±11 | 30±13 | N/A |
| VA→VA | Δ VA ↓ | -0.44±0.03 | -0.43±0.04 | -0.45±0.05 |
|  | Latency of Attenuation | 27±5.2 | 24±5.1 | 33±13 |
| A→V | A→V vs V_1_  Attenuating Neurons | -0.85±0.42  p=0.0486 | -0.87±0.52  p=0.1110 | -0.80±0.70  p=0.2844 |
| V→A | V→A vs A_1_  Attenuating Neurons | 0.61±0.35 p=0.1748 | 0.72±0.45 p=0.2074 | 0.54±0.51 p=0.3284 |
|  | V→A vs A_1_  Potentiating Neurons | 0.77±0.37 p=0.0602 | 0.87±0.38 p=0.0456 | 0.24±1.6  p=0.9047 |
| A→VA | A→VA vs VA_1_  Attenuating Neurons | -2.8±0.08 p<0.0001 | -2.9±0.11 p<0.0001 | -2.7±0.10 p=0.0020 |
| V→VA | V→VA vs VA_1_  Attenuating Neurons | -2.6±0.64 p=0.003 | -3.2±1.7 p=0.1585 | -2.2±0.41 p=0.0018 |
|  | V→VA vs VA_1_  Potentiating Neurons | 2.0±0.17 p<0.0001 | 2.0±0.19 p<0.0001 | 2.1±0.61 p=0.1772 |
| VA→A | VA→A vs A_1_ | -1.5±0.25 p<0.0001 | -1.7±0.34 p<0.0001 | -1.1±0.36 p=0.0055 |
| VA→V | VA→V vs V_1_ | -0.33±0.37 p=0.3714 | -0.05±0.36 p=0.8903 | -0.84±0.81 p=0.3145 |
